# Supplementary material for: A Combined Adjuvant TF–Al Consisting of TFPR1 and Aluminum Hydroxide Augments Strong Humoral and Cellular Immune Responses in Both C57BL/6 and BALB/c Mice
Source: Vaccines (Basel). 2021 Nov 29;9(12):1408. doi: 10.3390/vaccines9121408 (PMC8705145; doi:10.3390/vaccines9121408)
Supplement: Supplementary file 1 [file vaccines-09-01408-s001.zip › vaccines-1457142-supplementary.pdf]

Supplementary Materials

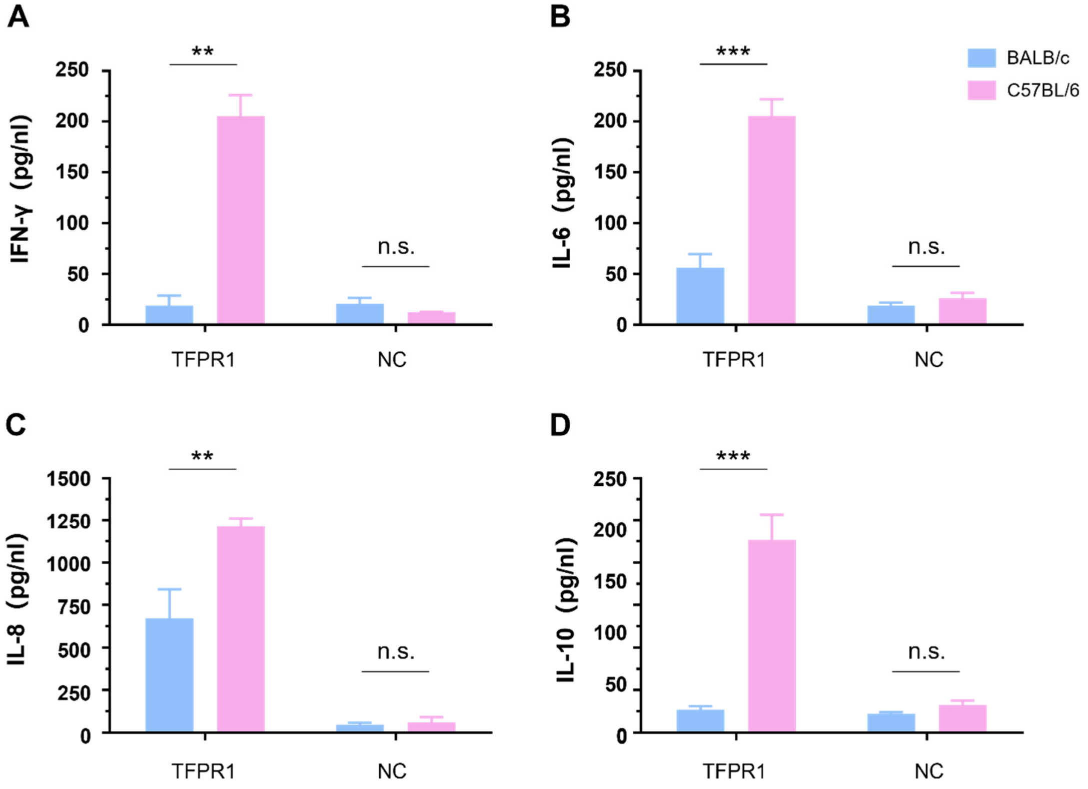

Figure S1. Detection of cytokine levels in the serum of BALB/c and C57BL/6 mice injected with TFPR1 for 3 h. BALB/c and C57BL/6 mice were immunized with TFPR1 intramuscularly and serum was collected after 3 h. The levels of cytokines were then measured using specific ELISA kits. (A) IFN-γ. (B) IL-6. (C) IL-8. (D) IL-10. Note: * stands for $p < 0.05$, ** $p < 0.01$, and *** $p < 0.001$.
